# A randomized controlled trial of a plastic-sheath technique to facilitate self-gripping mesh placement in laparoscopic inguinal hernia repair

**DOI:** 10.1007/s10029-026-03823-3

**Published:** 2026-08-20

**Authors:** Jakrapan Jirasiritham, Chairat Supsamutchai, Napaphat Poprom, Thanida Janbavonkij, Pongsasit Singhatat, Tharin Thampongsa, Jarinya Wilasrusmee, Theejutha Meakleartmongkol, Settanan Plangsiri, Panupol Thepyasuwan, Chumpon Wilasrusmee

**Affiliations:** 1 Department of surgery, Faculty of Medicine, Ramathibodi Hospital, Mahidol University, Rama VI Road, Bangkok, Thailand; 2 Department of surgery, Somdech Phra Pinklao Hospital, Bangkok, Thailand; 3 College of Medicine, Rangsit University, Pathum Thani, Thailand; 4 Faculty of Medicine Ramathibodi Hospital, Mahidol University, Bangkok, Thailand

**Keywords:** Self-gripping mesh, Inguinal hernia repair, Laparoscopic hernia repair, Total extraperitoneal (TEP) repairs, Surgical innovation

## Abstract

**Background:**

Self-gripping mesh reduces recurrence and postoperative pain but is difficult to deploy due to its adhesive properties.

**Methods:**

We developed a plastic-sheath covering technique to facilitate mesh placement. In Phase I, prototypes were evaluated for placement time and surgeon satisfaction. In Phase II, a randomized controlled trial (NCT04394338) compared the optimized sheath with conventional placement in laparoscopic inguinal hernia repair.

**Results:**

Prototype 4 achieved the shortest placement time in Phase I. In Phase II (*n* = 26), the plastic-sheath method significantly reduced mesh placement time (4.56 ± 1.80 vs. 7.40 ± 1.52 min; *P* = 0.005) and improved surgeon satisfaction (8.00 vs. 6.31; *P* = 0.003). Postoperative pain, hospital stay, and complication rates were comparable between groups.

**Conclusions:**

The plastic-sheath method simplifies self-gripping mesh deployment, reduces operative time, and improves surgeon satisfaction without increasing complications.

## Introduction

Groin hernias are among the most common surgical conditions worldwide. Standard treatment options include open repair with mesh placement using the Lichtenstein technique, as well as laparoscopic approaches such as total extraperitoneal (TEP) and transabdominal preperitoneal (TAPP) mesh repairs [1–6]. Recently, self-gripping mesh has been introduced to enhance outcomes in laparoscopic inguinal hernia repair. Research suggests that traumatic fixation of mesh may increase the risk of chronic postoperative inguinal pain (CPIP), making atraumatic fixation methods preferable.

Self-gripping mesh is a lightweight, monofilament polyester mesh with a semi-resorbable polylactic acid gripping system, enabling sutureless fixation to tissue. This design has been shown to result in low recurrence rates and minimal postoperative pain [7–11]. Additionally, its use eliminates the costs associated with mechanical fixation devices. However, deploying and positioning self-gripping mesh can be challenging due to its adhesive properties. Surgeons with substantial laparoscopic experience typically achieve proficiency in manipulating self-gripping mesh after 15 to 20 procedures [12].

In this study, we propose an innovative plastic covering technique to facilitate the placement of self-gripping mesh during laparoscopic hernia repair, addressing the challenges associated with its application and optimizing surgical outcomes.

## Materials and methods

In Phase I of the study, we developed innovative prototypes to simplify the placement of self-gripping mesh and address the challenges posed by its adhesive properties when unfolded in the operative field. This study was approved by the Institutional Review Board (IRB) of the Faculty of Medicine, Ramathibodi Hospital, Mahidol University (COA#MURA2020/184).

Our innovative approach utilized plastic sheaths, which we identified as a viable tool during the opening of the instrument port and mesh packaging. We adapted these sheaths for the mesh graft placement process. Initially, various configurations of plastic sheaths were designed and tested with the standard 9 cm × 15 cm self-gripping mesh. The meshes were then folded and unfolded over or under the plastic sheaths in different orientations.

**Fig. 1:**
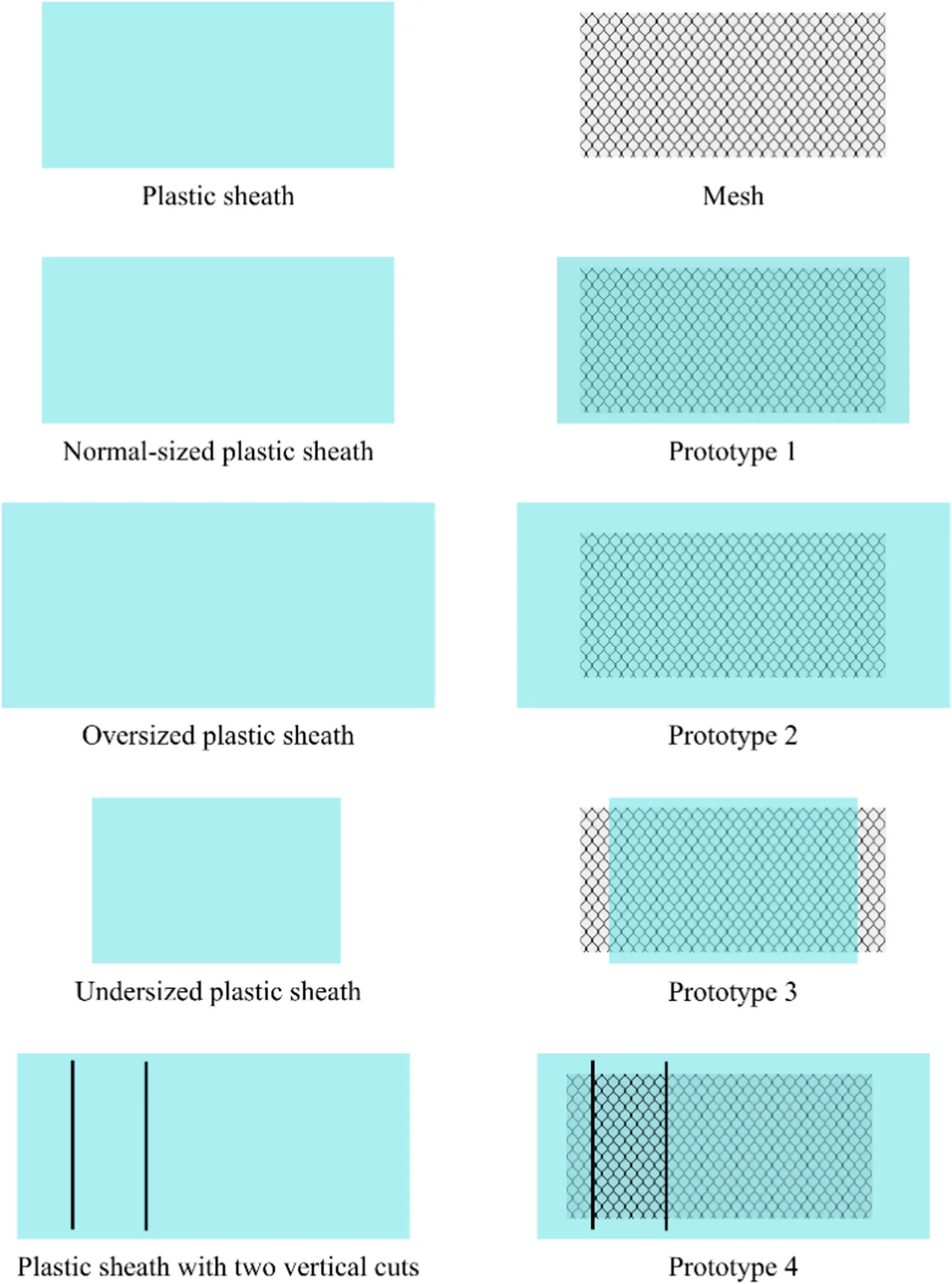
The mesh and plastic sheath preparation of prototype 1–4

From this testing, four prototypes were developed and evaluated for their suitability in clinical applications, see Fig. 1:

1. Prototype 1: A normal-sized plastic sheath measuring 9 cm × 15 cm used to cover the total area of the self-gripping mesh.
2. Prototype 2: An oversized plastic sheath measuring 10 cm × 16 cm designed to cover the total area of the self-gripping mesh.
3. Prototype 3: An undersized plastic sheath measuring 9 cm × 13 cm, covering the central area and leaving the medial and lateral edges of the mesh exposed.
4. Prototype 4: A plastic sheath measuring 10 cm × 15 cm with two vertical cuts created by 9 cm incisions positioned 1.5 cm and 3 cm from the medial edge. These incisions formed flaps that allowed the central non-gripping (bare) area of the mesh to remain exposed while the lateral portions remained covered by the sheath.

From January 2019 to September 2019, these prototypes were tested in patients undergoing elective laparoscopic groin hernia repair using the TEP technique and a 9 cm × 15 cm self-gripping mesh. In cases of bilateral inguinal hernias, one side was repaired using a plastic sheath prototype, while the other side employed the conventional technique without a sheath.

The time required for mesh deployment, with and without the use of plastic sheaths, was recorded for all prototypes. Surgeons assessed their satisfaction with the prototypes on a 0 to 10 analog scale, where 0 indicated complete dissatisfaction and 10 indicated maximum satisfaction. The mean satisfaction score for mesh placement without a plastic sheath was set at 5, serving as a baseline. Surgeons assigned scores after each operation. Finally, the mean deployment times and satisfaction scores were analyzed and reported for each prototype.

### Surgical procedure

The operation was performed under general anesthesia, with the patient positioned supine and both arms tucked securely. A Foley catheter was inserted in all cases prior to aseptic preparation of the operative field and was removed at the conclusion of the procedure. The TEP operation proceeded using the standard three-trocar technique. A 10-mm transverse sub-umbilical incision was made. The anterior rectus sheath was incised just lateral to the linea alba, and the rectus muscle was retracted laterally. The posterior aspect of the rectus muscle was then bluntly dissected from the posterior rectus sheath to create the initial preperitoneal space. To facilitate visualization, the operating table was adjusted to a slight head-down position.

A balloon dissector was inserted into the dissected space and inflated to expand the pre-peritoneal space in the lower abdomen. Subsequently, a blunt 10 mm trocar was inserted, and a 30-degree 10 mm laparoscopic camera was introduced. Two additional 5 mm trocars were placed midline between the umbilicus and the pubic bone. The pre-peritoneal space was further developed by dissecting loose connective tissue along the midline, extending down to the suprapubic bone. Key anatomical landmarks, including Cooper’s ligament and the iliopubic ligament, were identified. Lateral dissection continued to the level of the anterior superior iliac spine, exposing the hernia defect, cord structures, and epigastric vessels.

The hernia sac, whether direct or indirect, was carefully reduced to its origin. A self-gripping mesh, with or without a plastic sheath, was introduced into the pre-peritoneal groin area via the camera port. The mesh was vertically folded and positioned horizontally above the iliopubic tract. It was then unfolded centrally to cover the direct, indirect, and femoral hernia spaces adequately. After verifying the proper placement of the mesh, hemostasis and soft tissue integrity were assessed. The plastic sheath was removed via the lowest port along with the port trocar. Pneumoperitoneum was deflated, and all instruments were withdrawn. The anterior sheath at the subumbilical incision was closed with 2 − 0 Vicryl, and the skin was closed with 4 − 0 Vicryl using a subcutaneous suturing technique.

### Mesh and plastic sheath preparation

Sterile plastic sheaths of various sizes and configurations were prepared prior to the operation. The sheath consisted of a thin, flexible, translucent polymer film originally supplied as part of the sterile self-gripping mesh packaging. Before the start of surgery, the operating room scrub nurse prepared the selected sheath configuration and fitted the mesh into the plastic sheath under sterile conditions.

In prototypes 1 and 2, the gripping site of the mesh was aligned with the plastic sheath, and the edges along the long axis were rolled, leaving the plastic sheath on the outside before insertion into the preperitoneal space. In prototype 3, the mesh was prepared by rolling the bilateral edges of the long axis toward the center, creating vertical folds on both sides. Since the plastic sheath was smaller than the mesh, the medial and lateral edges of the mesh remained exposed. This exposed area allowed the mesh to be secured vertically. In prototype 4, the mesh was inserted into the vertical flap of the plastic sheath. A small, exposed area of the mesh served as the primary fixation point with the tissue before the mesh was fully unfolded, see Fig. 1.

### Mesh placement method

We inserted the mesh through the camera port into the preperitoneal space of the groin area. The mesh was positioned horizontally above the iliopubic ligament, extending from the medial to the lateral sides, and then unfolded vertically on both sides into the appropriate field.

For prototypes 1 and 2, after placing the mesh with its plastic sheath in the horizontal plane, the upper border of the mesh was anchored at two points from medial to lateral. Using a top-down placement technique, the plastic sheath was gradually pulled back downward to secure the mesh.

For prototype 3, the mesh with the plastic sheath was also placed horizontally. The adhesive area at the medial side was positioned and unfolded first, then fixed to the tissue in a bilateral vertical direction. The adhesive lateral portion was similarly unfolded, ensuring that the lateral part of the mesh remained unattached to the tissue while unrolling the plastic sheath. The mesh was progressively secured from medial to lateral as the plastic sheath was carefully removed.

**Fig. 2:**
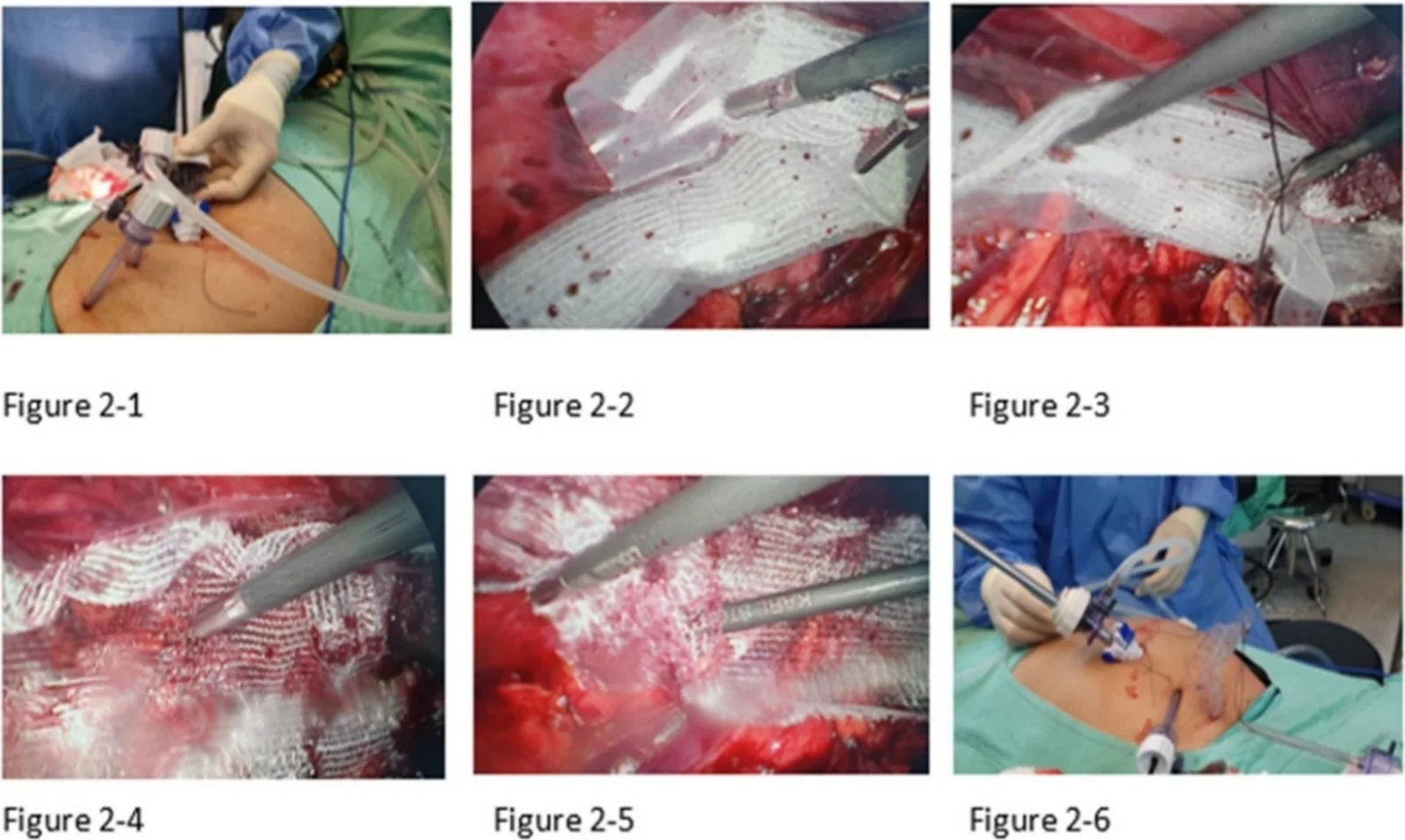
Mesh placement method of prototype 4

For prototype 4, after placing the mesh with the plastic sheath horizontally, the bare area, located 1.5 cm from the medial side, was initially anchored in its proper position. The mesh was then unfolded vertically on both sides and secured in place. The medial portion of the plastic sheath was pulled toward the medial side while attaching the medial part of the mesh to the tissue, leaving the sheath to lie over the non-gripping section of the mesh. Subsequently, the lateral side of the plastic sheath was gradually removed while simultaneously deploying the mesh. Once the entire mesh was securely placed and its position confirmed, the plastic sheath was removed via the lowest port, see Fig. 2.

### Randomized control trial to evaluate the efficacy of the plastic sheath

Phase II of the study involved a randomized controlled trial (NCT04394338) conducted from 16 January 2020 to 30 December 2022 to compare the novel plastic-sheath method with the conventional mesh placement technique during laparoscopic inguinal hernia repair. The study received approval from the Institutional Review Board of the Faculty of Medicine, Ramathibodi Hospital, Mahidol University (COA#MURA2020/184).

The study population consisted of patients aged 18 years and older who were scheduled for elective laparoscopic inguinal hernia repair using self-gripping mesh. Informed consent was obtained from all participants at the outpatient department before admission. Individuals who declined participation or withdrew consent were excluded from the study. Participants were randomly allocated to either the plastic-sheath group or the control group in a 1:1 ratio using a parallel assignment model. The allocation process was conducted through randomization generated by the Stata program.

The sample size for Phase II was estimated using mesh placement time as the primary outcome, based on the preliminary findings from Phase I. In Phase I, Prototype 4 reduced the mean mesh placement time from 6.29 min with the conventional method to 3.14 min, corresponding to an observed reduction of approximately 3.15 min. For the randomized phase, we used a more conservative assumption of a clinically meaningful difference of 2.5 min between groups, with a conservatively assumed standard deviation of 2.0 min. Using a two-sided alpha level of 0.05, 80% power, and an independent-samples t-test, the required sample size was approximately 11 patients per group. To account for possible dropout or incomplete data, the target sample size was increased to 13 patients per group, resulting in a total sample size of 26 patients.

**Fig. 3:**
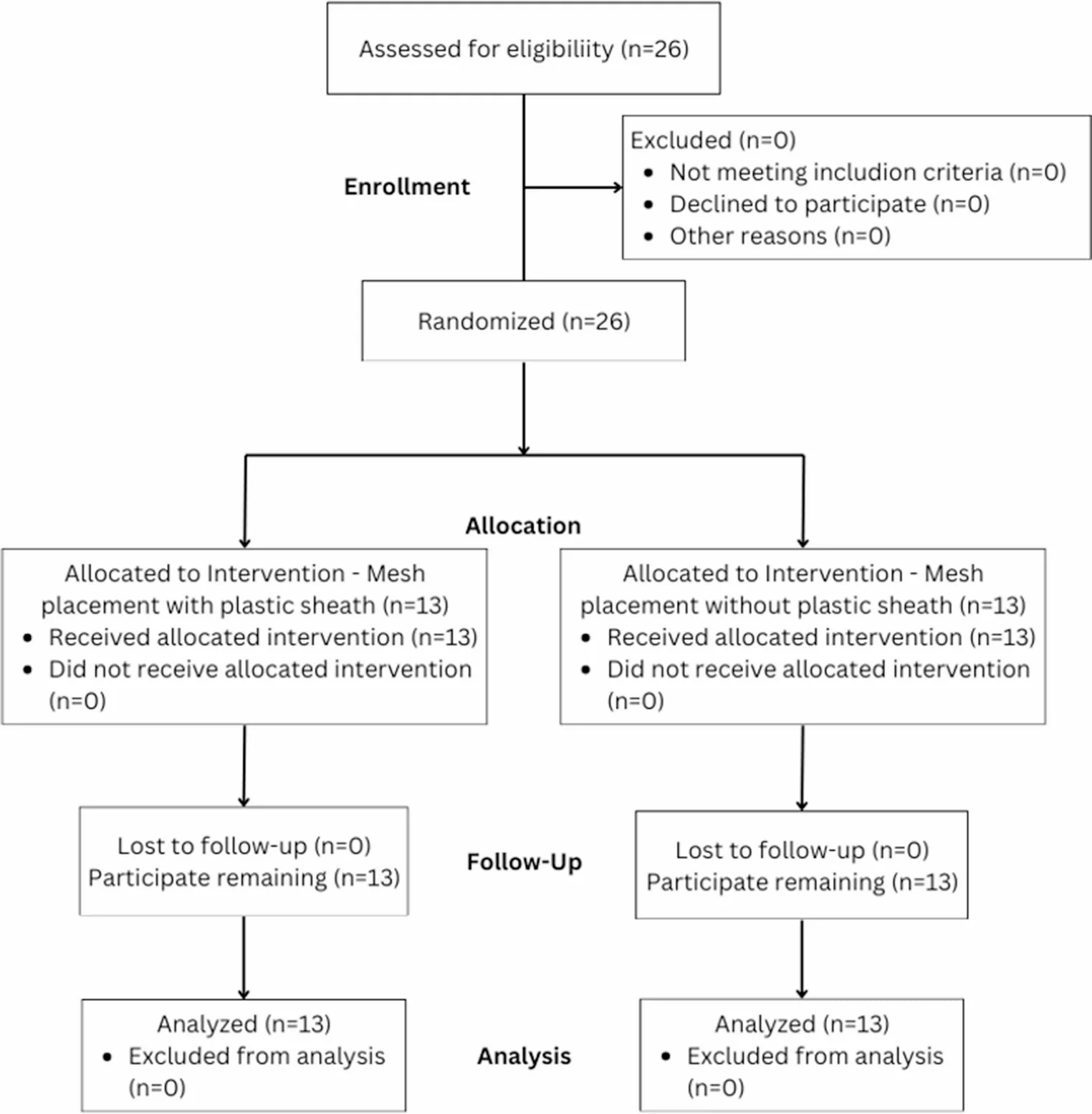
Consort participant flow diagram

In the intervention group, the plastic sheath (Prototype 4) was prepared in a specific configuration measuring 10 cm × 15 cm with vertical cuts at 1.5 cm and 3 cm from the edge. Meshes were unfolded over the plastic sheath to facilitate precise placement during the procedure. In the control group, meshes were placed conventionally without the use of a plastic sheath. There was no loss to follow-up, and data from all participants were included in the analysis. Refer to Fig. 3 for the participant flow diagram.

The study focused on the duration of mesh placement as the primary outcome, recorded in minutes from the introduction of the mesh to the completion of its placement. Secondary outcome included postoperative pain levels, measured 24 h after surgery using the Visual Analog Scale (VAS), and the occurrence of wound infections within 30 days. Surgeon satisfaction was assessed immediately after mesh placement using a subjective 10-point numeric rating scale, where 0 represented complete dissatisfaction and 10 represented maximal satisfaction. The score reflected the surgeon’s overall perception of the ease and convenience of mesh deployment. This assessment tool was not formally validated and was used as an exploratory measure in this study.

Statistical analysis was performed using the Chi-square test for categorical variables and Student’s 𝑡-test for continuous variables. A P-value of < 0.05 was considered statistically significant.

## Results

In Phase I, a total of 11 patients with inguinal hernias who underwent 18 laparoscopic TEP repairs were included for prototype testing. Among these, there were 4 cases of unilateral groin hernias and 7 cases of bilateral groin hernias, encompassing 10 right-sided and 8 left-sided hernias. The hernia types included 11 indirect inguinal hernias, 5 direct inguinal hernias, and 2 pantaloon hernias. No perioperative complications were observed.

Of the 18 TEP procedures, 7 were performed using the conventional method (mesh placement without a plastic sheath), while 9 used mesh placement with a plastic sheath. The latter group included 3, 3, 3, and 2 procedures with prototypes 1, 2, 3, and 4, respectively.

In terms of performance, the average time for mesh deployment using the conventional method was 6.29 min (SD 0.69). In comparison, the average mesh deployment times for prototypes 1, 2, 3, and 4 were 5.31 min (SD 0.54), 4.55 min (SD 0.23), 3.37 min (SD 1.01), and 3.14 min (SD 0.37), respectively. Surgeon satisfaction scores for the prototypes were progressively higher, with scores of 5, 6, 8, and 9 for prototypes 1, 2, 3, and 4, respectively, see Table 1.

**Table 1:** Plastic covering mesh prototype testing

Prototype	Sizes (cm)	Shapes	Placement time, Mean (± SD)	Satisfaction, (Scores)
1	9cmx15cm	Rectangle shape	5.31 (± 0.54)	5
2	10cmx16cm	Rectangle shape	4.55 (± 0.23)	6
3	9cmx13cm	Rectangle shape	3.37 (± 1.01)	8
4	10cmx15cm	Rectangle with two vertical cut	3.14 (± 0.37)	9

The outcomes were comparable between the conventional method and the plastic sheath prototypes in terms of hospital stay length and postoperative pain. During the follow-up period, no seroma, hematoma, wound infection, or early recurrence was reported.

Phase II randomized controlled trial enrolled a total of 26 patients undergoing the TEP procedure. The mesh placement time was significantly shorter in the plastic-sheath group, with an average duration of 4.56 min (SD 1.80) compared to 7.40 min (SD 1.52) in the conventional group (*P* = 0.005). Surgeon satisfaction scores were also notably higher in the plastic-sheath group, with a mean score of 8.0 (SD 0.63) compared to 6.31 (SD 0.98) in the conventional group (*P* = 0.003), see Table 2.

**Table 2:** Comparison between mesh placement with and without plastic-sheath

Outcome	Plastic-Sheath Group (*n* = 13) (Mean ± SD)	Control Group (*n* = 13) (Mean ± SD)	*P*-value
Mesh Placement Time (min)	4.56 ± 1.8	7.40 ± 1.52	0.005
Surgeon Satisfaction (0–10)	8.0 ± 0.63	6.31 ± 0.98	0.003
VAS Pain Score (0–10)	2.10 ± 0.80	2.30 ± 1.00	0.142
Wound Infections (%)	0%	0%	N/A

There were no significant differences in postoperative pain between the two groups. The mean VAS scores at 24 h post-surgery were similar, with a P-value of 0.142. Additionally, no wound infections were reported in either group within the 30-day postoperative period. Both groups also demonstrated comparable lengths of hospital stay, and no perioperative complications were observed throughout the trial.

## Discussion

The use of self-gripping mesh is one of several strategies to enhance outcomes and patient satisfaction in groin hernia operations, offering benefits such as safety, effectiveness, and reduced postoperative chronic pain. However, its adhesive properties can pose challenges, requiring a learning curve for surgeons. In this study, we present a novel method involving a plastic sheath to facilitate self-gripping mesh placement. This technique is straightforward, achieves high satisfaction, and significantly reduces placement time without complications. Among the prototypes tested, Prototype 4 was the most effective, with a mesh placement time of 3.14 min (2.88–3.40) and a satisfaction score of 9. Its design enabled efficient deployment by simplifying both the introduction process and proper positioning of the mesh, while minimizing the adhesive area in the medial site.

Previous studies have explored various techniques for self-gripping mesh placement. Bresnahan et al. [9] reported an average operative time of 66 min using a medial-to-lateral unfolding technique during TEP, with a mean mesh deployment time of 3.23 min (1.33–6.33). A horizontal bilateral unfolding method during TAPP and TEP, as described by Li et al. [13], achieved a mesh placement time of 2.83 min (0.75–4.17) and 3.27 min (0.75–4.00), respectively. Stavert et al. [11] presented a large retrospective series using self-gripping polyester mesh, demonstrating safety, effectiveness, and low recurrence rates with a rolling technique. Muysoms et al. [14] reported a mesh placement time of 5.20 min (± 5.10) during laparoscopic inguinal hernia repair but did not specify the placement technique.

In our study, the bilateral vertical unfolding technique without a plastic sheath yielded a mean mesh deployment time of 6.29 min (4.88–7.08), consistent with findings from Muysoms et al. [14]. Variations in reported times across studies may stem from differences in timing calculation methods, mesh designs, placement techniques, or surgeon experience. For instance, delays in our study were occasionally due to improper initial placement requiring repositioning.

The introduction of a plastic sheath improved mesh placement times across all prototypes. The sheath prevented premature adhesion to surrounding tissues during insertion, allowing for quicker and more precise placement. Despite some challenges, such as increased bulk during insertion and occasional obstruction of the operative field, the plastic sheath generally enhanced the deployment process.

Prototype 4 demonstrated superior performance compared with the other prototypes, which may be attributable to several design characteristics. In Prototypes 1 and 2, the plastic sheath covered the entire non-gripping surface of the mesh. While this configuration facilitated insertion of the mesh into the preperitoneal space by preventing premature adhesion, removal of the sheath required it to be withdrawn from beneath the deployed mesh. This additional manipulation could lead to displacement of the mesh and necessitate repositioning, thereby increasing deployment time.

Prototypes 3 and 4 were designed to overcome this limitation by leaving a portion of the mesh exposed. The exposed area allowed the mesh to be anchored to the target tissue before complete removal of the sheath, helping to maintain the desired position during deployment. We speculate that although Prototype 3 benefited from this anchoring effect, the exposed medial and lateral edges remained susceptible to unintended adhesion to surrounding tissues during placement, which may have contributed to delays and reduced ease of handling.

In contrast, Prototype 4 exposed only a central portion of the mesh while keeping the lateral edges covered by the plastic sheath. This design may have reduced the likelihood of unintended adhesion while still allowing the mesh to be anchored securely at the intended position. Furthermore, the central anchoring point may have improved stability during sheath removal, facilitating more controlled deployment and contributing to the shorter placement times and higher surgeon satisfaction observed with this prototype.

The randomized controlled trial comparing Prototype 4 with conventional mesh placement further demonstrated the effectiveness of this approach, showing a significant reduction in mesh placement time and improved surgeon satisfaction. Importantly, no postoperative infections or procedure-related complications were observed. Additionally, preparation of the sheath was performed by the nursing team, while the clinical procedures, data collection, and outcome assessments were performed by the operating surgeons. All participating surgeons were attending surgeons at the Faculty of Medicine Ramathibodi Hospital and had prior experience with self-gripping mesh placement.

The study has several limitations. First, because the Phase II study enrolled only 26 patients, the sample size was calculated to provide sufficient statistical power to detect differences in mesh placement time but may not have been adequate to detect differences in other outcomes, such as surgical complications. Second, this was a single-center study, which may limit the generalizability of the findings. Furthermore, because the intervention was visible to the surgeons, blinding was not feasible, potentially introducing both observer and performance bias. The surgeons’ level of experience with mesh placement should also be considered, as all participating surgeons were already familiar with self-gripping mesh placement. Consequently, the findings may not be directly generalizable to less experienced surgeons. Finally, the 30-day follow-up period may have been insufficient to detect long-term outcomes, including late complications and recurrence. Further studies with larger sample sizes, multicenter designs, and longer follow-up periods are warranted to confirm these findings and better evaluate long-term outcomes.

## Conclusion

We introduced a novel plastic sheath covering method for self-gripping mesh placement, featuring a design with two vertical cuts. This method is straightforward, achieves high satisfaction, and significantly reduces placement time by preventing adhesion to surrounding tissues before proper placement. Moreover, no complications were observed. This innovative approach has the potential to shape the future of inguinal hernia repair surgery, offering a safer and more efficient alternative for surgeons.

## Data Availability

The datasets used and analyzed during the current study is available from the corresponding author on reasonable request.
