# Correction to: A randomized controlled trial of a plastic-sheath technique to facilitate self-gripping mesh placement in laparoscopic inguinal hernia repair

**DOI:** 10.1007/s10029-026-03871-9

**Published:** 2026-10-01

**Authors:** Jakrapan Jirasiritham, Chairat Supsamutchai, Napaphat Poprom, Thanida Janbavonkij, Pongsasit Singhatat, Tharin Thampongsa, Jarinya Wilasrusmee, Theejutha Meakleartmongkol, Settanan Plangsiri, Panupol Thepyasuwan, Chumpon Wilasrusmee

**Affiliations:** 1 Department of surgery, Faculty of Medicine, Ramathibodi Hospital, Mahidol University, Rama VI Road, Bangkok, Thailand; 2 Department of Surgery, Somdech Phra Pinklao Hospital, Bangkok, Thailand; 3 College of Medicine, Rangsit University, Pathum Thani, Thailand; 4 Faculty of Medicine Ramathibodi Hospital, Mahidol University, Bangkok, Thailand

**Correction to: Hernia (2026) 30:323**

**https://doi.org/10.1007/s10029-026-03823-3**

In the section named” Randomized control trial to evaluate the efficacy of the plastic sheath” of the original article, the study period was incorrectly mentioned as “16 January 2020 to 30 December 2022”.

It should read as

The study was conducted from 16 January 2020 to 18 April 2022.

